# Genome sequence of endophyte *Pseudomonas* sp. Can09R isolated from the roots of *Chenopodium album* L.

**DOI:** 10.1128/mra.00466-26

**Published:** 2026-07-31

**Authors:** Vladimir K. Chebotar, Maria S. Gancheva, Margarita V. Khudyaeva, Oksana V. Keleinikova, Maria E. Baganova, Alexander N. Zaplatkin, Veronika N. Pishchik, Elena P. Chizhevskaya

**Affiliations:** 1 All-Russia Research Institute for Agricultural Microbiology, Pushkin, St. Petersburg, Russia; 2 Saint Petersburg State University 48544, St. Petersburg, Russia; University of Strathclyde, Glasgow, United Kingdom

**Keywords:** genome sequence, endophyte, *Pseudomonas mercuritolerans*, roots, *Chenopodium album*

## Abstract

Endophyte *Pseudomonas* sp. Can09R was isolated from surface-sterilized roots of *Chenopodium album* L. DNA sequencing was performed using the Illumina platform, and the genome was assembled with SPAdes software. The final scaffolded assembly spans 6,036,228 bp, has a guanine–cytosine content of 60%, and contains 5,281 predicted protein-coding genes.

## ANNOUNCEMENT

The endosphere of drought-tolerant plants, such as *Chenopodium album* L., represents a promising reservoir of beneficial bacteria for agriculture. The strain Can09R was isolated from the roots of *C. album* L. collected in the Stavropol region in Russia (N 44°33′43″, E 44°49′59″). An average root sample from three plants (5 g) was cleaned to remove soil, rinsed with tap water, surface-sterilized by sequential immersion in tap water (30 s), 70% ethanol (5 min), 15% H₂O₂ (10 min), and five 2-min sterile water rinses, and then crushed with a sterile pestle and mortar in 2.5 mL of 0.85% NaCl. The homogenate (0.5 mL) was streaked onto R2A medium (Thermo Fisher Scientific, Waltham, MA, USA), and cultured at 28°C for 72 h. The strain was isolated from a separate colony after re-streaking on R2A medium. Strain Can09R was grown in R2A medium at 30°C overnight for DNA extraction. DNA was extracted from a single colony using the Wizard Genomic DNA Purification Kit (Promega, USA), and 100 ng of extracted DNA was used for library preparation with the KAPA HyperPlus Kit (Roche, Switzerland). Library sizing was performed with the High-Sensitivity DNA Kit (Agilent Technologies, USA). Sequencing was conducted on the NextSeq 1000 platform (Illumina, USA) using NextSeq 1000/2000 P1 Reagents (300 cycles) with a 2% PhiX spike-in control. A total of 1,769,174 read pairs were generated (2× 150 bp).

The quality of the raw reads was assessed using FastQC v0.11.9 (1), which indicated high-quality data with no need for additional trimming or filtering. Genome assembly was performed using SPAdes v3.14.1 (2) and resulted in 69 contigs (*N*50 = 674,953 bp). FastANI v1.34 (3) against all *Pseudomonas* reference genomes downloaded from National Center for Biotechnology Information (NCBI) RefSeq (4) (accessed May 2026) showed the highest similarity of Can09R to *Pseudomonas mercuritolerans* strain SAICEUPSM (GCF_025706525.1) with an average nucleotide identity of 98.12%. Scaffolding was performed by aligning contigs to the reference genome with Ragtag v2.1.0 (5), followed by removal of scaffolds shorter than 500 bp only if they were not aligned to the reference genome. Genome coverage was calculated using the samtools coverage tool v1.21 (6) by mapping reads to the assembly with BWA v0.7.18 (7). Assembly quality metrics were evaluated with QUAST v5.1.0 (8), CheckM analysis v1.2.4 (*Pseudomonas* marker set) (9), and BUSCO v5.2.2 (gammaproteobacteria_odb10) (10). The final assembly consisted of 11 scaffolds totaling 6,036,228 bp, with a guanine–cytosine (GC) content of 60%, an average sequencing depth of 87.5×, and an *N*50 of 928,087 bp. BUSCO and CheckM analyses confirmed 99.8 and 99.63% completeness of the assembly, respectively. The genome annotation was carried out using the NCBI Prokaryotic Genome Annotation Pipeline v6.10 (11), resulting in the identification of 5,413 genes, of which 5,281 are protein-coding. Unless otherwise specified, default software parameters were applied.

Based on its isolation from surface-sterilized roots of *C. album* L., Can09R is presumed to be an endophyte. Similar to the type strain SAICEUPSMT, which promotes plant growth under mercury stress (12), Can09R may enhance plant performance under drought conditions, although this hypothesis requires further experimental validation.

## Data Availability

The genome sequence and raw reads were deposited in National Center for Biotechnology Information and can be accessed under BioProject PRJNA1439611 (SRA SRR37687833, assembly GCA_056333215.1).
